# An Abdominal Presentation of Churg-Strauss Syndrome

**DOI:** 10.1155/2010/290654

**Published:** 2010-08-10

**Authors:** J. R. E. Rees, P. Burgess

**Affiliations:** ^1^Department of General Surgery, Gloucestershire Royal Hospital, Great Western Road, Gloucester, GL1 3NN, UK; ^2^Department of General Surgery, Great Western Hospital, Marlborough Road, Swindon, SN3 6BB, UK

## Abstract

Churg-Strauss syndrome is a small and medium vessel vasculitis that is also known as allergic granulomatous angiitis. It most commonly presents with an asthma like symptoms. It was first described in Mount Siani Hospital, New York in 1951 by Jacob Churg and Lotte Stauss and was recognised after the study of a series of 13 patients who had asthma, eosinophilia, granulomatous inflammation necrotising systemic vasculitis and necrotising glomerulonephritis. We describe a case of Churg-Strauss syndrome presenting with abdominal pain and later during the hospital admission a mono-neuritis multiplex syndrome affecting the lower limbs. The patient presented in such an atypical fashion with abdominal signs and symptoms that they required laparotomy and the diagnosis was made after histological examination of tissue taken at the time of surgery. Treatment with immunosuppression and aggressive rehabilitation achieved a progressive recovery which continued on discharge from hospital.

## 1. Background

Churg-Strauss syndrome is a small and medium vessel vasculitis and is also known as allergic granulomatous angiitis. It affects small and medium size arteries and veins and is closely related to both Wegners granulomatosis and the microscopic form of periarteritis (microscopic polyangitis). It is associated with perinuclear antineutrophil cytoplasmic antibody (p-ANCA) positivity in up to 40%–50% of cases [1].

The syndrome was first described in Mount Siani Hospital, New York in 1951 by Churg and Strauss [2]. It was recognised after the study of a series of 13 patients who had asthma, eosinophilia, granulomatous inflammation necrotising systemic vasculitis, and necrotising glomerulonephritis. It was further described by Zeek in 1952 [3] as an allergic granulomatous angiitis of a necrotic type and Zeek specifically suggested that it differed from hypersensitivity vasculitis. It presents with a broad range of local and systemic manifestations and is believed to have three phases. Initially individuals have an asthma type illness often with allergic rhinitis, this then progresses to conditions such as pneumonia and gastroenteritis which are associated with eosinophilic infiltration. Finally a small and medium vessel vasculitis arises with associated chronic granulomatous inflammation. This may be marked by specific end organ damage, for example, renal, cardiac, pulmonary, dermatological, and very commonly a mononeuritis multiplex.

We present a case of Churg-Strauss syndrome presenting with abdominal pain and later during the hospital admission a mononeuritis multiplex syndrome affecting the lower limbs. 

## 2. Case Report

A 44-year-old man was assessed at our institution after emergency referral by his general practitioner with a one week history of left-sided abdominal and flank pain with pain spreading to the left thigh. There was a history of a fever-like illness and some diarrhoea. He was apyrexial at initial presentation however a fever developed later. His past history consisted only of mild asthma managed with metered dose steroid and bronchodilator inhalers and allergic rhinitis. There was tenderness on palpation in the left iliac fossa and left flank and straight leg raise on the left intensified the pain. Initial investigations showed a raised white count and a raised CRP of more than 100 mg/L but normal renal and hepatic function. He underwent chest and abdominal radiography which showed loss of the left psoas shadow but normal abdominal gas pattern and no pneumoperitoneum.

An initial diagnosis of acute diverticulitis with an associated inflammation or abscess within the left psoas was made. Intravenous access was established; intravenous fluids, analgesia and intravenous Co-Amoxiclav 1.2 g tds, and Gentamicin 5 mg/kg OD were administered.

**Figure 1:**
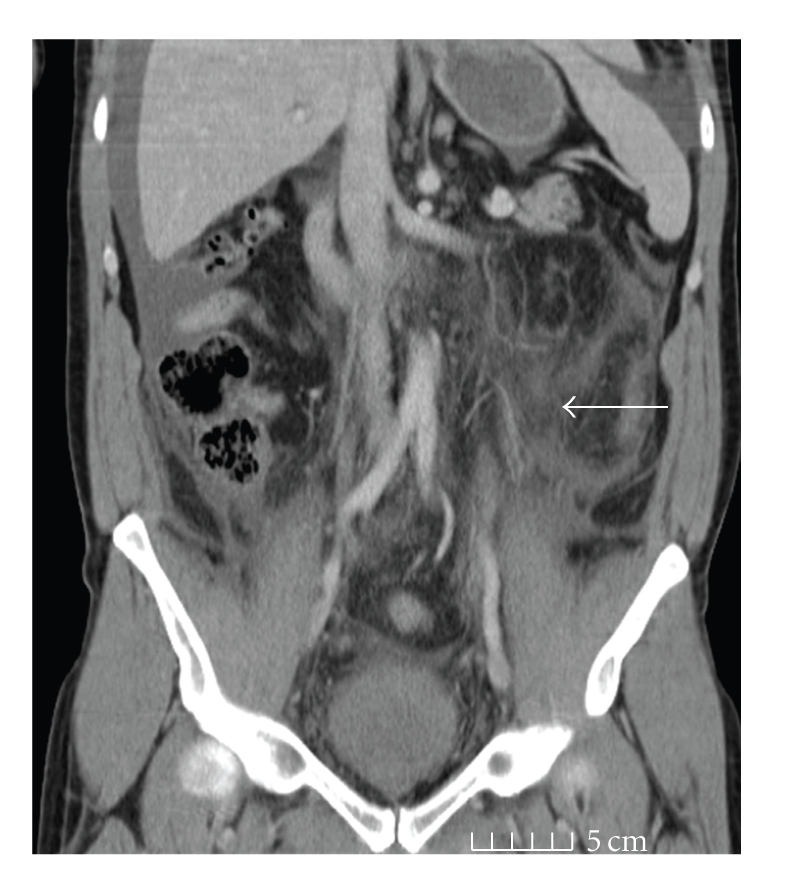
Coronal computerised tomography image showing diffuse inflammation affecting the peritoneum of the left side of the abdomen, the pelvis and the left psoas and retroperitoneum. (Marked by white arrow).

The day after admission his pain had worsened particularly in the left thigh and increased weakness was noted in the left thigh. At this point a CT of the abdomen and pelvis was performed. This showed diffuse inflammation affecting the peritoneum of the left side of the abdomen, the pelvis, and the left psoas and retroperitoneum, but no collection was seen (Figure 1). The following day the lower limb neurological symptoms worsened with numbness affecting the L1/L2 distribution, quadriceps weakness on the left, and similar weakness on the right side. An MRI of the thoracolumbar and sacral spine was performed and an opinion sought from the neurology service. The MRI did not reveal any significant abnormality of the spine or spinal nerve roots and confirmed the presence of inflammation affecting the left psoas, left sided retroperitoneum, and associated left sided abdominal and pelvic peritoneum. Repeat haematological investigations at this stage revealed an eosinophilia that peaked at 4.52 × 10^9^/L (Normal range 0–0.5 × 10^9^/L). The diffuse inflammation raised the possibility of inflammatory bowel disease and a colonoscopy was performed at this time; however, the colon was both macroscopically normal and random biopsies showed it to be microscopically normal. 

At this stage ANA, p-ANCA, and c-ANCA, cryoglobulins, complement studies, hepatitis screen, and serum protein electrophoresis were all normal.

Over the following 24 hours his abdominal symptoms and signs dramatically worsened and he became peritonitic; concern about the possibility of mesenteric ischaemia was raised and based on the clinical findings we proceeded to laparotomy. 4 litres of ascites were immediately identified at this point, which was thought to have arisen from a diffuse inflammatory peritoneal response and associated hypoalbuminaemia producing an ascitic transudate. The ascites were drained and a sample sent for cytological examination. Further examination of the abdominal cavity revealed diffuse inflammatory infiltrate particularly involving the appendix, small bowel, peritoneum, and omentum. An appendicectomy was performed along with sampling of the small bowel, peritoneum, and greater omentum. 

**Figure 2:**
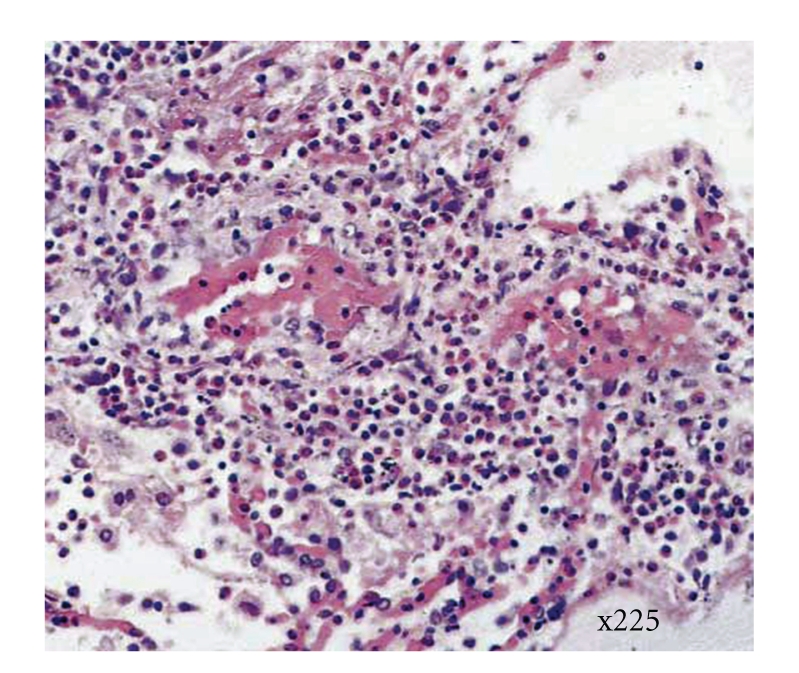
High-powered image of haematoxylin and eosin-stained section of a vessel with eosinophilic infiltrate and vascular distortion, x225 magnification.

He recovered slowly, but there was continued persistence of his neurological symptoms and pain. Cytology of the ascitic fluid showed numerous eosinophils, with histiocytes and reactive mesothelial cells whilst histology from the surgical samples which became available three days later showed tissue with diffuse serosal coverage by an extensive eosinophilic exudate which extended into the subserosa of the excised appendix with an associated subserosal focal eosinophilic vasculitis. H&E histological staining of tissue containing vessels with eosinophilic infiltrate and distortion is shown in Figure 2. Within the omental tissue in addition to the eosinophilic infiltrate, occasional multinuclear giant cell were seen. No mycobacteria, parasites, or other infective organisms were seen.

These findings were strongly suggestive of Churg-Strauss and a specialist opinion was sought from the Rheumatology service at our institution. Pulsed intravenous methyl prednisolone 1 g tds was started and a plan made, once a full recovery from the surgery had occurred, to convert to oral prednisolone 50 mg per day (reducing dose regime) and cyclophosphamide 750 mg iv together with prophylactic Mesna 400 mg before and after the cyclophosphamide. This was initially planned at 2 weekly intervals and then monthly according to response with regular monitoring of the platelet count. 

His recovery was unfortunately delayed by wound infection and then a wound dehiscence requiring resuturing under general anaesthesia. Therefore, although the steroid therapy was initiated immediately cyclophosphamide was delayed until 4 weeks after the abdominal resuturing. His quadriceps weakness slowly improved and a minor improvement in sensation in the L1 and L2 distributions were identified. He transferred to a rehabilitation facility 37 days after admission and 34 days after his laparotomy and was discharged home after 48 days. A reducing dose regime of oral prednisolone was prescribed on discharge, starting at 50 mg/day and reducing by 5 mg per week and plans for a reducing frequency cyclophosphamide infusion made. On review at 6 weeks his surgical wounds had healed, strength in his quadriceps had increased, and sensation in the L1 and L2 dermatomes had almost returned to normal.

## 3. Discussion

Churg-Strauss syndrome is rare and is defined by six criteria [4]: (1) bronchial asthma; (2) Eosinophilia >10% by differential white cell count; (3) Mono- or polyneuropathy; (4) Nonfixed pulmonary infiltrates on chest radiography; (5) paranasal sinus abnormalities; (6) Biopsy containing blood vessels with extravascular eosinophils [5, 6]. Presence of 4 out of 6 of these criteria has 99.7% specificity and an 85% sensitivity for Churg-Strauss [6]. 

Our patient had four out of the six criteria described; the only features he did not exhibit were nonfixed pulmonary infiltrates on chest radiography. We did not investigate him for paranasal sinus abnormalities, but he did have allergic rhinitis. The peritoneal, omental, and appendiceal biopsies did show extravascular eosinophilic infiltrates and in addition multinucleate giant cells were seen consistent with the most advanced phase of Churg-Strauss syndrome in the omental tissue sampled [7]. 

Churg-Strauss syndrome presents with a wide range of symptoms, asthma affects nearly all individuals (97%) and may precede the vasculitis by up to 10 years, nearly two thirds (61%) of individuals have sinusitis; allergic rhinitis is also common but necrotizing lesions (typical of Wegners granulomatosis) are, however, rare but polyposis is common. Cough and haemoptysis occur in just over a third of individuals (37%); arthralgias occur in 40% of patients, whilst skin changes occur in half of all patients (49%). Cardiac events may also occur and rarely Churg-Strauss may affect the eyes or even cause stroke.

Churg-Strauss manifests in the gastrointestinal tract in 31% of cases, causing abdominal pain as in our case, diarrhoea, and occasionally gastrointestinal bleeding [8, 9]. The need for laparotomy is rare; in this case it might be hypothesised that the severe local inflammation generated significant peritoneal irritation, producing signs initially local and then diffusing peritonism. The dehiscence is an additional unfortunate complication in our case and likely reflects, overall poor nutritional state, high-dose steroid therapy, and perhaps the systemic inflammatory effect of the underlying vasculitis. 

Neuropathy is also common. There is commonly a mononeuritis multiplex (77% of cases) but may be a polyneuropathy as became apparent in this case. 

There is a significant eosinophilia, and often an anaemia; ESR and CRP are raised; p (perinuclear)-ANCA is positive in 40%–50% of cases; serum IgE may be raised and there is often a hypergammaglobulinaemia. Rheumatoid factor may be weakly positive and there are elevations in eosinophils cationic protein, soluble interleukin-2 receptor (sIL-2R), and soluble thrombomodulin which specifically mark endothelial injury [10, 11].

Histopathologically Churg-Strauss syndrome is characterised by small necrotizing granulomas, and necrotizing vasculitis involving the small and medium vessels. The granulomas consist of a central eosinophilic core surrounded radially by macrophages and epitheliod giant cells, which were a feature of our case [7].

Treatment involves immunosuppression and begins with steroids; these may be oral if the manifestations are mild; however these should be intravenous if there is major organ involvement or neuropathy. Approximately 30% of patients require more aggressive immunosuppression; these are individuals who have severe disease or have failed to respond to steroids. This is initially with cyclophosphamide and is typically once weekly, and as a clinical response is detected the frequency of administration is reduced [12].

In addition, immunosuppression with azathioprine [13], mycophenlotae [14] and intravenous immunoglobulin [15] have also been used. A small number of cases of treatment with interferon alpha have been reported but the true efficacy remains unclear [16].

More recently a number of reports have suggested immunomodulation using the genetically modified anti-CD20 monoclonal antibody rituximab, and the chimeric monoclonal anti-TNF*α* antibody infliximab may offer benefits in Churg-Strauss, in refractory cases and also by either avoiding the use of cytotoxic agents or reducing steroid dependency [17, 18].

In the long term this systemic vasculitis has a 90% 1 year survival and a 62% 5 years survival, without treatment the survival is significantly diminished with 5 year survival being only 25% [19]. Relapse is common, and long-term steroid therapy is often required; nasal involvement often marks a relatively good prognosis in Churg-Strauss syndrome, which is in contrast to Wegner's Granulomatosis whilst gastrointestinal tract disease, as demonstrated in our case, often marks a poorer long term outcome and increased risk of relapse [20]. 

In summary we present a diagnostically challenging and atypical case of Churg-Strauss syndrome presenting with abdominal and flank pain and a polyneuropathy which required extensive investigation and laparotomy to achieve a diagnosis.

## Abbreviations

ANA: Antinuclear antibody
c-ANCA: C-terminus antineutrophil cytoplasmic antibodies
CRP: C-Reactive protein
CT: Computerised tomography
ESR: Erythrocyte sedimentation rate
MRI: Magnetic resonance imaging
OD: Omni die (once a day)
p-ANCA: Myeloperoxidase anti neutrophil cytoplasmic antibodies
TDS: Ter die sumendus (three times a day)
H&E: Haematoxylin and eosin.

## References

[1] Pagnoux C, Guillevin L (2010). Churg-Strauss syndrome: evidence for disease subtypes?. *Current Opinion in Rheumatology*.

[2] Churg J, Strauss L (1951). Allergic granulomatosis, allergic angiitis, and periarteritis nodosa. *The American Journal of Pathology*.

[3] Zeek PM (1952). Periarteritis nodosa; a critical review. *American Journal of Clinical Pathology*.

[4] Suzuki M, Nabeshima K, Miyazaki M, Yoshimura H, Tagawa S, Shiraki K (2005). Churg-Strauss syndrome complicated by colon erosion, acalculous cholecystitis and liver abscesses. *World Journal of Gastroenterology*.

[5] Nishie M, Tomiyama M, Kamijo M (2003). Acute cholecystitis and duodenitis associated with Churg-Strauss syndrome. *Hepatogastroenterology*.

[6] Masi AT, Hunder GG, Lie JT (1990). The American College of Rheumatology 1990 Criteria for the classification of Churg-Strauss syndrome (allergic granulomatosis and angiitis). *Arthritis and Rheumatism*.

[7] Lie JT, Hunder GG, Arend WP (1990). Illustrated histopathologic classification criteria for selected vasculitis syndromes. *Arthritis and Rheumatism*.

[8] Singh R, Singh D, Abdou N (2009). Churg-Strauss syndrome presenting as acute abdomen: are gastrointestinal manifestations an indicator of poor prognosis?. *International Journal of Rheumatic Diseases*.

[9] Pagnoux C, Mahr A, Cohen P, Guillevin L (2005). Presentation and outcome of gastrointestinal involvement in systemic necrotizing vasculitides: analysis of 62 patients with polyarteritis nodosa, microscopic polyangiitis, Wegener granulomatosis, Churg-Strauss syndrome, or rheumatoid arthritis-associated vasculitis. *Medicine*.

[10] Keogh KA, Specks U (2003). Churg-Strauss syndrome: clinical presentation, antineutrophil cytoplasmic antibodies, and leukotriene receptor antagonists. *American Journal of Medicine*.

[11] Schmitt WH, Csernok E, Kobayashi S, Klinkenborg A, Reinhold-Keller E, Gross WL (1998). Churg-strauss syndrome: serum markers of lymphocyte activation and endothelial damage. *Arthritis and Rheumatism*.

[12] Lanham JG, Elkon KB, Pusey CD, Hughes GR (1984). Systemic vasculitis with asthma and eosinophilia: a clinical approach to the Churg-Strauss syndrome. *Medicine*.

[13] Ribi C, Cohen P, Pagnoux C (2008). Treatment of Churg-Strauss syndrome without poor-prognosis factors: a multicenter, prospective, randomized, open-label study of seventy-two patients. *Arthritis and Rheumatism*.

[14] Assaf C, Mewis G, Orfanos CE, Geilen CC (2004). Churg-Strauss syndrome: successful treatment with mycophenolate mofetil. *British Journal of Dermatology*.

[15] Taniguchi M, Tsurikisawa N, Higashi N (2007). Treatment for Churg-Strauss syndrome:induction of remission and efficacy of intravenous immunoglobulin therapy. *Allergology International*.

[16] Tatsis E, Schnabel A, Gross WL (1998). Interferon-*α* treatment of four patients with the Churg-Strauss syndrome. *Annals of Internal Medicine*.

[17] Kaushik VV, Reddy HV, Bucknall RC (2006). Successful use of rituximab in a patient with recalcitrant Churg-Strauss syndrome. *Annals of the Rheumatic Diseases*.

[18] Tiliakos A, Shaia S, Hostoffer R, Kent L (2004). The use of infliximab in a patient with steroid-dependent churg-strauss syndrome. *Journal of Clinical Rheumatology*.

[19] Guillevin L, Lhote F, Gayraud M (1996). Prognostic factors in polyarteritis Nodosa and Churg-Strauss syndrome: a prospective study in 342 patients. *Medicine*.

[20] Pavone L, Grasselli C, Chierici E (2006). Outcome and prognostic factors during the course of primary small-vessel vasculitides. *Journal of Rheumatology*.

